# Individual and community level determinants of minimum meal frequency among breastfeeding children aged 6–23 months in Ethiopia: a multilevel analysis of 2019 Ethiopian Demographic Health Survey data

**DOI:** 10.3389/fpubh.2024.1445370

**Published:** 2024-11-15

**Authors:** Dagnachew Melak, Eyob Tilahun Abeje, Fekade Demeke Bayou, Fekadeselassie Belege Getaneh, Lakew Asmare, Abel Endawkie, Alemu Gedefie, Amare Muche, Anissa Mohammed, Aznamariam Ayres

**Affiliations:** ^1^Department of Epidemiology and Biostatistics, School of Public Health, College of Medicine and Health Sciences, Wollo University, Dessie, Ethiopia; ^2^Department of Pediatrics and Child Health Nursing, College of Medicine and Health Sciences, Wollo University, Dessie, Ethiopia; ^3^Department of Medical Laboratory Science, College of Medicine and Health Sciences, Wollo University, Dessie, Ethiopia

**Keywords:** minimum meal frequency, EMDHS 2019, breastfeeding children, mixed effect analysis, Ethiopia

## Abstract

**Background:**

Minimum meal frequency (MMF) is a vital indicator of whether a child’s energy needs are being met. Previous studies in Ethiopia on MMF have primarily focused on individual factors, often using basic logistic regression models with limited scope and small sample sizes. In contrast, this study aims to identify the key determinants of MMF among breastfed children aged 6–23 months in Ethiopia, examining both individual and community-level factors through multilevel logistic regression analysis, utilizing nationally representative data.

**Method:**

The study utilized a community-based cross-sectional design with a sample of 1,245 6–23 month breastfed children in Ethiopia, using data from the 2019 Ethiopian Mini Demographic Health Survey. Mixed effect logistic regression was used to identify factors associated with MMF. Adjusted odds ratios (AOR) with 95% confidence intervals were used to assess variable associations, while Intra-cluster correlation (ICC), median odds ratio (MOR), and proportional change in variance (PCV) were employed to gauge random variation.

**Result:**

The proportion of 6–23-month breastfeeding children with MMF was found to be 55.9% (95% CI: 53.2, 58.6). In mixed effect logistic regression; breastfeeding initiation time within days after birth [AOR = 0.44; 95%CI: (0.24, 0.80)], maternal primary educational level [AOR = 1.46; 95%CI: (1.03, 2.05)], maternal higher level education [AOR = 2.43; 95%CI: (1.22, 4.81)] and children from wealthiest household wealth index [AOR = 2.13; 95%CI: (1.04, 4.33)] were individual-level factors and children from city-based regions [AOR = 1.75; 95%CI: (1.10, 2.77)] was community level factors significantly associated with MMF. The final model indicates that 50.8% of the variation in MMF was explained by combined factors at both individual and community levels and also the variation across clusters remained statistically significant (ICC = 8.1% and variance = 0.29 with *p* < 0.001).

**Conclusion:**

Minimum meal frequency is a proxy for meeting energy requirements and it is a key indicator of infant and young child feeding (IYCF) practice. According to this study finding, the prevalence of MMF among 6–23-month children in Ethiopia was found to be low. The study also suggest that time of breastfeeding initiation, women’s educational level, household wealth index, and contextual region were factors significantly associated with MMF. It is recommended that IYCF strategies should be implemented widely through women’s education and empowerment and promoting early initiation of breast feeding to increase the proportion of children to be fed with MMF appropriate for their age.

## Introduction

The World Health Organization (WHO) recommends exclusive breastfeeding for the first 6 months of a child’s life, followed by the introduction of nutritionally safe and adequate complementary foods until the age of two and older (1, 2). Children between 6 and 23 months face a higher risk of under nutrition as they transition from their mother’s milk to solid foods, leading to potential issues like indigestion, infection, and insufficient food (3, 4).

Ensuring proper nutrition in infancy and early childhood is crucial for the optimal growth, health, and development of children (5). For breastfed children aged 6–8 months, meeting the minimum meal frequency involves getting solid, semisolid, or soft foods at least twice a day. Meanwhile, breastfed children aged 9–23 months should have such foods at least three times a day to meet the minimum meal frequency (2, 4, 6).

Minimum meal frequency (MMF) and Minimum Acceptable Diet (MAD) serve as key indicators of whether a child’s energy needs are being met (2, 7). Ensuring a minimum meal frequency is crucial for the healthy growth and development of infants and children aged 6–23 months. Without sufficient dietary diversity and meal frequency, young children become vulnerable to malnutrition, including stunting, micronutrient deficiencies, growth faltering and an increased risk of illness and mortality (1, 2). Infants indeed require higher levels of nutrition and energy due to their rapid growth, but they do not consume larger quantities less frequently for several key reasons related to their physical development, digestion, and nutritional needs (6). In Africa, a minority of 6 to 23-month-old children meet the minimum standards for dietary diversity (less than one-third) and meal frequency (around 50%) (8). In addition, less than 20% of children receive sufficient complementary feeding (9). This inadequate child feeding is likely to be a contributing factor to the high prevalence of stunting in many African countries, as emphasized in a recent review of stunting risk factors in sub-Saharan Africa (10). Moreover, improper complementary feeding practices elevate the likelihood of under nutrition, illness, and mortality in infants and children under 2 years old (11). Surviving malnourished children face an elevated risk of morbidity and endure long-term consequences of malnutrition, potentially affecting subsequent generations (12).

According to the 2019 Ethiopian Mini Demographic Health Survey (EMDHS), only 11% of children aged 6–23 months meet the minimum acceptable diet across all three IYCF practices, while 14% have a sufficiently diverse diet (i.e., consuming foods from the appropriate food groups), and 55% were fed the minimum required number of times for their age (2).

Even though the government of Ethiopia tried to promote optimal feeding practices for 6–23-month-old children, there is still a poor improvement (2). This could be due to several contributing factors including low household wealth index (4), the low level of maternal educational status (13–15), household size (16), low Media exposure of mothers (13, 14) low growth monitoring program (GMP) utilization (13), maternal employment (15–18), low antenatal and postnatal visits (17, 19), and poor counseling of mother on optimal child feeding practice (19). Furthermore, minimum meal frequency could also be influenced by the community-level factors including place of residence, contextual region, community level of maternal education, community level of antenatal care (ANC) and postnatal care (PNC) utilization, community-level of employment, and community level of media exposure (16, 20, 21). Barriers such as cultural practices, lack of knowledge, social norms, beliefs, household decision-making dynamics, competing responsibilities, and limited access to nutrient-rich foods are all contributing to the deterioration of women and children’s nutritional status (1).

Previous studies in Ethiopia on minimum meal frequency primarily focused on individual-level factors, utilizing basic logistic regression models with limited scope and small sample sizes (4, 13, 15, 17–19) and another study only address determinants of minimum acceptable diet (MAD) without separately analyze factors particularly influence MMF (22). However, MMF can be influenced by both individual and community-level factors, as individual behavior toward meal frequency is shaped not only by personal factors but also by community-level influences, with infants nested within communities. The assumption of independence among individuals within the same cluster and equal variance across clusters is violated in nested data scenarios. Given the hierarchical nature of EMDHS data and the violation of the independence assumption in basic logistic regression, accounting for clustering effects is preferred. Therefore, this current study aims to identify determinants of MMF among breastfed children aged 6–23 months in Ethiopia, considering both individual and community levels factors, using multilevel logistic regression analysis. The study’s findings are intended to offer valuable insights for program managers and policymakers, helping them design and implement targeted interventions to address inadequate meal frequency. These efforts aim to reduce childhood malnutrition, illness, and mortality in Ethiopia.

## Materials and methods

### Study area, study design, and study period

This study was done in Ethiopia, situated in North-Eastern Africa, this study took place in a country characterized by agrarian, pastoralist, and urban populations (2). The majority, 83.6%, reside in rural areas, while 16.7% live in urban areas. Ethiopia has a fertility rate of 6.7, an infant mortality rate of 47 deaths per 1,000 live births, and a child mortality rate of 59 deaths per 1,000 live births in 2019 EMDHS (2). A community-based cross-sectional study was conducted among breastfeeding children aged 6–23 months. The survey was conducted from March 21, 2019, to June 28, 2019.

### Study participants

The research encompassed all youngest children born within 2 years before the survey in the chosen clusters during the 2019 EDHS data collection period. The study focused on breastfed children aged 6–23 months residing in selected clusters within EDHS enumeration areas, specifically those present at least one night before data collection, as the study population.

### Data source, sample size determination and sampling technique

This study utilized secondary data from the 2019 EMDHS, obtained with approval from Measure Demographic health survey (DHS) and downloaded from https://dhsprogram.com. The 2019 EMDHS sample selection involved two stages and was stratified by region into urban and rural areas, creating 21 sampling strata. Enumeration Areas (EAs) were chosen independently in each stratum in two stages, using implicit stratification and proportional allocation. In the first stage, 305 EAs were selected with probability proportional to EA size. Household listing was conducted in all selected EAs, with larger EAs (over 300 households) segmented to reduce listing efforts. In the second stage, 30 households per cluster were chosen through equal probability systematic selection from the household listing. No replacements or changes to pre-selected households were allowed to prevent bias. Eligibility for the survey were all women aged 15–49 in selected households or those who spent the night before the survey in those households. The survey covered 9,150 residential households (2,790 urban, 6,360 rural), aiming for 7,959 completed interviews with women aged 15–49 (2,636 urban, 5,323 rural) and 4,825 children aged 0–59 months with height and weight measurements (852 urban, 3,973 rural). For the current study, 1,245 (weighted) last-born children aged 6–23 months and currently breastfeeding were included (2).

### Variables of the study and operational definition

The study focused on the dependent variable of minimum meal frequency among breastfed children aged 6–23 months in Ethiopia. For breastfed children aged 6–8 months, meeting the minimum meal frequency was considered if the child received solid, semisolid, or soft foods at least twice daily. Meanwhile, breastfed children aged 6–23 months are deemed to meet the minimum meal frequency if they receive solid, semisolid, or soft foods at least three times a day otherwise they were considered as “not meet the minimum meal frequency (2, 7).

Individual-level predictors encompassed child-related factors (child’s age, sex, birth order, birth interval, and breastfeeding initiation time), maternal socio-demographic factors (mother’s age, sex of household head, marital status, education, household wealth index, media exposure, number of under-five children and family size), and obstetric and healthcare-related variables (ANC and PNC utilization, place and mode of delivery, delivery assistance, and parity). Additionally, community-level variables considered in the study included place of residence, contextual region, community media exposure, community wealth index, community women’s education, community ANC utilization, and community PNC utilization.

Community-level variables were created by consolidating individual characteristics within a cluster since EMDHS did not directly collect data describing cluster characteristics, apart from the place of residence and regions. The aggregates were calculated using the proportions of subcategories for a given variable. As the aggregate values for all generated variables were not normally distributed, they were grouped based on national median values and previous relevant studies (23–25).

Community ANC utilization refers to the percentage of mothers in a specific cluster who attended ANC a certain number of times. It was classified into three categories based on national quartiles: low ANC utilization communities (where ≤25% of women utilize ANC), middle (when 25–75% of women utilize ANC), and high (when >75% of women utilize ANC) (23). The community level of PNC utilization represents the percentage of women in a specific cluster who attend PNC a certain number of times. It was divided into two categories: low (when <50% of women utilized PNC) and high (when ≥50% of women utilized PNC) (20, 21, 23).

The community-level media exposure refers to the combined exposure of respondents within a community to various media types, categorized as “Low” (<50% media exposure), and “High” (≥50% exposure) media-utilized communities (23). The community-level wealth index is an aggregated wealth index categorized as “Low” (<25% wealthy), “Moderate” (25–50% wealthy), and “High” (>50% wealthy) communities (23–25).

Ethiopia is administratively divided into 11 regions for contextual purposes. These regions are classified based on the living conditions of the population as agrarian, pastoralist, or city-based. Regions such as Tigray, Amhara, Oromia, south nation and nationality of people (SNNP), Gambella, and Benshangul Gumuz are categorized as agrarian. Somali and Afar regions are grouped to form the pastoralist region, while Harari region, Addis Ababa, and Dire Dawa city administrations are grouped to represent city-based populations (2, 23).

The community level of women’s education denotes the percentage of women in a community with at least primary education, categorized as low (when ≤50% of women were educated), and high (when >50% of women were educated) (23–25).

### Data processing and analysis

Extracted variables from the EMDHS 2019 dataset underwent coding, recoding, and exploratory analysis. Categorization was performed for continuous variables, and data were managed, cleaned, and weighted for analysis using STATA version 17. Descriptive measures like mean, percentage, graphs, and frequency tables were used to characterize the study population. The multilevel analysis involved four models to determine the best-fit model: Model one (Null model) assessed cluster-level differences in minimum meal frequency, Model two adjusted for individual variables, Model three assessed community-level factors, and Model four included both adjusted individual and community-level factors. The log probability of minimum meal frequency was modeled using a two-level multilevel model. Log [ $\frac{\pi_{ij}}{1-\pi_{ij}}$ ] = $\beta_{0}$ + $\beta_{1}X_{ij}+\beta_{2}Z_{ij}+u_{j}+e_{ij}$ (24, 25).

Where, *i* and *j* represent individual (level 1) and clusters (level 2) units, respectively. The variables *X* and *Z* pertain to individual and community-level variables, respectively. The symbol π_ij_ denotes the probability of minimum meal frequency for the *i*th child in the *j*th community, and the *β*’s represent the fixed coefficients. Specifically, β_0_ serves as the intercept, signifying the impact on the probability of minimum meal frequency when predictors have no influence. The term *u*_j_ reflects the random effect, indicating the influence of the community variable on minimum meal frequency for the *j*th community, while *e*_ij_ accounts for random errors at the individual level. By assuming that each community has a distinct intercept (*β*_0_) and fixed coefficient (*β*), the analysis considers the clustered nature of the data and accommodates variations within and between communities.

Multilevel logistic regression was employed for data analysis due to the hierarchical nature of DHS data. This modeling addresses unexplained variation in minimum meal frequency, capturing unobserved cluster factors as random effects. All models featured a random intercept at the cluster level to account for heterogeneity among clusters. The fixed-effects estimated the association between various explanatory variables and the likelihood of a child achieving minimum meal frequency, expressed as Adjusted Odds Ratios (AOR) with 95% Confidence Intervals (CI). Crude associations were initially examined independently, and variables with *p* ≤ 0.2 in the bi-variable analysis were included in the multivariable analysis model. In multivariable analysis, variables with *p* ≤ 0.05 and a confidence interval excluding the null value were considered statistically significant for minimum meal frequency.

In this multi-level logistic regression analysis, four models were sequentially constructed. The first model, termed the Null model, lacked predictors. Model two encompassed only individual-level factors, while model three focused solely on community-level factors. Finally, model four integrated both individual and community-level factors. Variables demonstrating a statistically significant association with minimum meal frequency in the final model were selected based on a *p*-value of 0.05 and odds ratios not including the null value.

Measures of variation, such as Intra-cluster Correlation (ICC), Median Odds Ratio (MOR), and Proportional Change in Variance (PCV), were reported. ICC explained cluster variation, while MOR measured unexplained cluster heterogeneity. The ICC indicated the variation in minimum meal frequency among children aged 6–23 months attributed to community characteristics. A higher ICC (>5%) suggested greater relevance of community characteristics in understanding individual variation in minimum meal frequency. [ICC= $\frac{\;\delta^{2}u}{\delta^{2}u+\delta^{2}e}$ ] where *δ*^2^*u* = between-group variation, *δ*^2^*e* = within-group variation, or [ICC= $\frac{\;\delta^{2}}{\delta^{2}+\frac{\pi^{2}}{3}}$ ] where *δ*^2^ is the estimated variance of clusters (24, 25).

The Median Odds Ratio (MOR) is the median value of the odds ratio between the areas with the highest and lowest likelihood of minimum meal frequency, chosen randomly. It gauges unexplained cluster heterogeneity, indicating the variation between clusters by comparing two individuals from two randomly selected different clusters. MOR can be computed using a specific formula [MOR = exp ( $\sqrt{2x\delta^{2}+0.6745}$ ) ≈ exp (0.95 δ )] (24).

In this study, MOR indicates how much an individual’s likelihood of minimum acceptable meal frequency is influenced by their residential area. The Proportional Change in Variance (PCV), calculated as ((*V*_0_ – *V*_1_)/*V*_0_)*100, where *V*_0_ is the variance of the initial model and *V*_1_ is the variance of the model with additional terms, measures the total variation attributed by individual and community factors in the multilevel model. PCV was computed for each model, using the null model as a reference to demonstrate the explanatory power of the factors in explaining minimum meal frequency. The goodness of fit for the adjusted final model, compared to previous models (individual and community level), was assessed using a Log-likelihood test, Deviance Information Criteria (DIC), and Akaike Information Criteria (AIC). The model with the highest Log likelihood value and the lowest DIC and AIC values were considered the best-fit model.

### Ethical consideration

Ethical clearance was not required since secondary data was taken from DHS but permission to access the dataset was granted by the Measure DHS International Program. The data, used solely for this study, remained confidential and was not shared with any third party. The information utilized in this research was anonymous, publicly available, and aggregated secondary data, devoid of personal identity. The complete dataset was accessible on the DHS website.^1^

## Results

### Children related characteristics

This research involved examining the minimum meal frequency among a group of 1,203 last-born children aged 6–23 months who were currently being breastfed. The data was drawn from the 2019 EMDHS. After adjusting for sample weight, the total sample size became 1,245, all of whom were included in the analysis. The mean age of the children was 14.3 months (SD ± 5.01), and approximately 648 (52%) of them were male. The majority of children, around 918 (74%), had begun breastfeeding immediately after birth (Table 1).

**Table 1:** Characteristics of breastfeeding children aged 6–23 months with their cross-tabulation of minimum meal frequency in Ethiopia, 2019 (*N* = 1,245).

Variables	Categories	Weighted frequency (%)	Minimum meal frequency	
			Yes (%)	No (%)
Child age	6–8 months	241 (19.35%)	121 (50.2%)	120 (49.8%)
	9–23 months	1,004 (80.64%)	574 (57.2%)	430 (42.8%)
Sex of child	Male	648 (52%)	360 (55.6%)	288 (44.4%)
	Female	597 (48%)	336 (56.3%)	261 (43.7%)
Birth order	First	311 (24.98%)	200 (64.3%)	111 (35.7%)
	2–3	449 (36.06%)	241 (53.7%)	208 (46.3%)
	4–5	252 (20.24%)	151 (59.9%)	101 (40.1%)
	6+	233 (18.71%)	103 (44.2%)	130 (55.8%)
Breastfeeding initiation time	Immediately	918 (73.73%)	539 (58.7%)	379 (41.3%)
	Within hours	274 (22.01%)	139 (50.7%)	135 (49.3%)
	Within days	53 (4.26%)	17 (32.1%)	36 (67.9%)
Birth interval (preceding)	No previous birth	311 (24.98%)	200 (64.3%)	111 (35.7%)
	<24 months	168 (13.49%)	81 (48.2%)	87 (51.8%)
	≥24 months	766 (61.53%)	414 (54.0%)	352 (46.0%)

### Maternal socio-demographic characteristics

The majority of mothers 605 (48.59%) with breastfeeding children aged 6–23 months fell within the age range of 25–34 years. During the survey period, 1,187 (95.34%) of the mothers were in a union, and the majority 534 (42.89%) had no formal education. In terms of the number of under-five children in their households, about 686 (55.10%) mothers had 2–3 children, and 465 (37.35%) lived in households with a family size of ≤4 persons. Concerning the household wealth index, approximately 282 (22.65%) of the respondents were categorized in poorer households (Table 2).

**Table 2:** Maternal socio-demographic characteristics with their cross-tabulation of minimum meal frequency of children in Ethiopia, 2019 (*N* = 1,245).

Variables	Categories	Weighted frequency (%)	Minimum meal frequency	
			Yes (%)	No (%)
Mothers age	15–24 years	413 (33.17%)	254 (61.5%)	159 (38.5%)
	25–34 years	605 (48.59%)	312 (51.6%)	292 (48.4%)
	35–49 years	227 (18.23%)	129 (56.8%)	98 (43.2%)
Sex of household head	Male	1,081 (86.83%)	605 (56.0%)	476 (44.0%)
	Female	164 (13.17%)	90 (54.9%)	74 (45.1%)
Marital status	Currently in union	1,187 (95.34%)	667 (56.2%)	520 (43.8%)
	Not in union	58 (4.66%)	29 (50.0%)	29 (50.0%)
Educational status	No education	534 (42.89%)	255 (47.7%)	279 (52.3%)
	Primary	531 (42.65%)	318 (59.9%)	213 (40.1%)
	Secondary	112 (7.00%)	73 (65.2%)	39 (34.8%)
	Higher	68 (5.46%)	49 (72.1%)	19 (27.9%)
Wealth index	Poorest	225 (18.07%)	80 (35.6%)	145 (64.4%)
	Poorer	282 (22.65%)	148 (52.5%)	134 (47.5%)
	Middle	241 (19.36%)	143 (59.3%)	98 (40.7%)
	Richer	237 (19.04%)	140 (59.1%)	97 (40.9%)
	Richest	260 (20.88%)	185 (71.2%)	75 (28.8%)
Media exposure	No	814 (65.38%)	434 (53.3%)	380 (46.7%)
	Yes	431 (34.62%)	261 (60.6%)	170 (39.4%)
Number of under 5 children	0–1	550 (44.18%)	337 (61.3%)	213 (38.7%)
	2–3	686 (55.10%)	353 (51.5%)	333 (48.5%)
	4–5	9 (0.72%)	5 (55.6%)	4 (44.4%)
Family size	≤4	465 (37.35%)	288 (61.9%)	177 (38.1%)
	5–6	413 (33.17%)	225 (54.5%)	188 (45.5%)
	7–8	239 (19.20%)	125 (52.3%)	114 (47.7%)
	≥9	128 (10.28%)	58 (45.3%)	70 (54.7%)
Religion	Christianity	837 (67.23%)	450 (53.8%)	387 (46.2%)
	Muslim	392 (31.48%)	242 (61.7%)	150 (38.3%)
	Others	16 (1.28%)	3 (18.8%)	13 (81.2%)

### Maternal obstetric and healthcare-related characteristics

In terms of maternal ANC service utilization, 988 (79.36%) mothers had attended ANC visits during their last pregnancy. Among them, approximately 711 (57.11%) had given birth at a health facility, with around 72 (5.78%) undergoing cesarean delivery. Skilled birth attendants, including doctors, nurses, midwives, health officers, and health extension workers, assisted in about 731 (58.70%) of the deliveries (Table 3).

**Table 3:** Maternal obstetric and healthcare-related characteristics with their cross-tabulation of minimum meal frequency of children in Ethiopia, 2019 (*N* = 1,245).

Variables	Categories	Weighted frequency (%)	Minimum meal frequency	
			Yes (%)	No (%)
ANC utilization	No	257 (20.64%)	119 (46.3%)	138 (53.7%)
	Yes	988 (79.36%)	577 (58.4%)	411 (41.6%)
PNC utilization	No	1,156 (92.85%)	637 (55.1%)	519 (44.9%)
	Yes	89 (7.15%)	58 (65.2%)	31 (34.8%)
Place of delivery	Home	534 (42.89%)	262 (49.1%)	272 (50.9%)
	Health facility	711 (57.11%)	434 (61.0%)	277 (39.0%)
Delivery by CS	No	1,173 (94.22%)	661 (56.4%)	512 (43.6%)
	Yes	72 (5.78%)	35 (48.6%)	37 (51.4%)
Delivery assistance	Non-skilled birth attendants	514 (41.30%)	254 (49.4%)	260 (50.6%)
	Skilled birth attendants	731 (58.70%)	441 (60.3%)	290 (39.7%)
Parity	Nulliparous	311 (24.98%)	200 (64.3%)	111 (35.7%)
	Multiparous	934 (75.02)	495 (53.0%)	439 (47.0%)

### Community level determinants of minimum meal frequency in Ethiopia

In this study, the majority of residents 898 (72.13%) lived in rural areas, and approximately 1,127 (90.52%) of the respondents belonged to agrarian communities. Most respondents 804 (64.58%) hailed from communities with a high proportion of wealth. Regarding women’s education within the community, around 680 (54.62%) were from communities with a high proportion of educated women. Similarly, approximately 681 (54.69%) women came from communities with high levels of ANC utilization (Table 4).

**Table 4:** Community-level determinants of minimum meal frequency of children in Ethiopia, 2019 (*N* = 1,245).

Variables	Categories	Weighted frequency (%)	Minimum meal frequency	
			Yes (%)	No (%)
Place of residence	Urban	347 (27.87%)	201 (57.9%)	146 (42.1%)
	Rural	898 (72.13%)	494 (55.0%)	404 (45.0%)
Contextual region	Agrarian	1,127 (90.52%)	637 (56.5%)	490 (43.5%)
	Pastoralist	73 (5.86%)	24 (32.9%)	49 (67.1%)
	City-based	45 (3.61%)	34 (75.5%)	11 (24.4%)
Community media exposure	Low	864 (69.39%)	473 (54.7%)	391 (45.3%)
	High	381 (30.60%)	223 (58.5%)	158 (41.5%)
Community wealth index	Low	276 (22.17%)	121 (43.8%)	155 (56.2%)
	Moderate	165 (13.25%)	85 (51.5%)	80 (48.5%)
	High	804 (64.58%)	489 (60.8%)	315 (39.2%)
Community-women education	Low	565 (45.38%)	282 (49.9%)	283 (50.1%)
	High	680 (54.62%)	413 (60.7%)	267 (39.3%)
Community ANC utilization	Low	48 (3.85%)	18 (37.5%)	30 (62.5%)
	Middle	516 (41.44%)	249 (48.3%)	267 (51.7%)
	High	681 (54.69%)	429 (63.0%)	252 (37.0%)
Community PNC utilization	Low	98 (7.87%)	68 (69.4%)	30 (30.6%)
	High	1,147 (92.13%)	627 (54.7%)	520 (45.3%)

### Prevalence of minimum meal frequency among 6–23 month children in EMDHS

In this study; out of 1,245 breastfeeding 6–23 month age children, 55.9% with 95% CI (53.2, 58.6) of children were fed with minimum meal frequency standard appropriate for age and their breast feeding status.

### Determinants of minimum meal frequency among 6–23 month breastfeeding children in EMDHS

A bi-variable mixed-effect logistic regression was conducted to identify potential variables for inclusion in the multivariable multilevel logistic regression model. Consequently, the following variables were considered as candidates for inclusion in the multivariable analysis based on a significance level of *p*-value ≤0.2: age and sex of the child, birth order, timing of breastfeeding initiation, birth interval, mother’s age and sex of household head, education level, household wealth index, maternal media exposure, number of under-five children in the household, family size, ANC service utilization during the last pregnancy, place of delivery, cesarean section delivery, type of delivery assistance, parity, place of residence, contextual region, community media exposure, community wealth index, community women’s education, community ANC service utilization, and community PNC service utilization.

As depicted in Table 5, the final model highlighted several statistically significant associations with minimum meal frequency. Specifically, breastfeeding initiation time, mothers’ educational level, household wealth index, and contextual region exhibited notable association. Notably, breastfeeding initiation time displayed a negative association with minimum meal frequency. Children who commenced breastfeeding within days had a 56% lower likelihood [AOR = 0.44; 95%CI: (0.24, 0.80)] of achieving minimum meal frequency compared to those initiated immediately after birth.

**Table 5:** Mixed effect logistic regression analysis for individual and community level determinant of minimum meal frequency among 6–23 month breastfeeding children in Ethiopia, 2019 (*N* = 1,245).

Variables	Categories	Model one	Model two AOR (95%CI)	Model three AOR (95%CI)	Model four AOR (95%CI)
Child age	6–8 months		1		1
	9–23 months		1.23 (0.89, 1.69)		1.23 (0.89, 1.69)
Sex of child	Male		1		1
	Female		1.15 (0.89, 1.50)		1.12 (0.86, 1.45)
Birth order	First		1		1
	2–3		0.76 (0.48, 1.19)		0.81 (0.51, 1.27)
	4–5		0.90 (0.49, 1.64)		0.93 (0.52, 1.69)
	6^+^		0.69 (0.35, 1.38)		0.75 (0.38, 1.48)
Breastfeeding initiation time	Immediately		1		1
	Within hours		0.78 (0.56,1.07)		0.78 (0.56, 1.08)
	Within days		0.44 (0.24, 0.80)*		0.44 (0.24, 0.80)***
Mothers age	15–24 years		1		1
	25–34 years		0.92 (0.63, 1.34)		0.88 (0.61, 1.27)
	35–49 years		1.54 (0.88, 2.69)		1.46 (0.83, 2.55)
Sex of household head	Male		1		1
	Female		1.28 (0.9,1.81)		1.12 (0.86,1.45)
Mother education	No education		1		1
	Primary		1.47 (1.05, 2.04)*		1.46 (1.03, 2.05)*
	Secondary		1.50 (0.87, 2.58)		1.45 (0.84, 2.52)
	Higher		2.21 (1.12, 4.36)*		2.43 (1.22, 4.81)**
HH wealth index	Poorest		1		1
	Poorer		1.49 (0.98, 2.27)		1.35 (0.87, 2.11)
	Middle		1.56 (1.01, 2.42)*		1.39 (0.84, 2.30)
	Richer		1.95 (1.19, 3.18)*		1.69 (0.96, 2.97)
	Richest		2.31 (1.36, 3.91)*		2.13 (1.04, 4.33)*
Media exposure	No		1		1
	Yes		1.09 (0.77, 1.52)		0.99 (0.69, 1.43)
Number of under 5 children	0–1		1		1
	2–3		0.99 (0.71, 1.38)		0.98 (0.70, 1.36)
	4–5		2.56 (0.78, 8.39)		2.66 (0.82, 8.65)
Family size	≤4		1		1
	5–6		1.02 (0.71, 1.48)		1.02 (0.71, 1.47)
	7–8		0.90 (0.56, 1.42)		0.88 (0.56, 1.39)
	≥9		0.70 (0.41, 1.21)		0.72 (0.42, 1.24)
ANC utilization	No		1		1
	Yes		1.59 (1.11, 2.27)*		1.39 (0.97, 2.02)
Place of delivery	Home		1		1
	Health facility		1.38 (0.59, 3.23)		1.18 (0.51, 2.74)
Delivery by CS	No		1		1
	Yes		1.11 (0.63, 1.96)		1.05 (0.59, 1.83)
Delivery assistance	Skilled birth attendant		1		1
	Non-skilled birth attendant		1.52 (0.65, 3.54)		1.49 (0.65, 3.44)
Place of residence	Urban			1	1
	Rural			1.21 (0.76, 1.93)	1.47 (0.85, 2.55)
Contextual region	Agrarian			1	1
	Pastoralist			0.99 (0.62, 1.59)	1.19 (0.72, 1.96)
	City-based			1.68 (1.09, 2.57)*	1.75 (1.10, 2.77)*
Community media exposure	Low			1	1
	High			0.99 (0.62, 1.60)	0.80 (0.48, 1.34)
Community wealth index	Low			1	1
	Moderate			1.75 (1.09, 2.82)	1.52 (0.90, 2.57)
	High			1.70 (1.13, 2.56)*	1.25 (0.76, 2.07)
Community-women education	Low			1	1
	High			1.17 (0.83, 1.65)	1.03 (0.71, 1.49)
Community ANC utilization	Low			1	1
	Middle			1.24 (0.63, 2.45)	1.01 (0.49, 2.04)
	High			2.42 (1.17, 4.99)*	1.82 (0.84, 3.98)
Community PNC utilization	Low			1	1
	High			0.66 (0.38, 1.15)	0.72 (0.40, 1.28)

*p < 0.05, **p < 0.01, ***p < 0.001.

Maternal educational level exhibited a positive association with minimum meal frequency, with mothers possessing a primary education being 1.46 times more likely [AOR = 1.46; 95%CI: (1.03, 2.05)], and those with higher education 2.43 times more likely [AOR = 2.43; 95%CI: (1.22, 4.81)], to adhere to minimum meal frequency compared to mothers without formal education. Similarly, the household wealth index demonstrated a positive association, as children from the wealthiest households were 2.13 times more likely [AOR = 2.13; 95%CI: (1.04, 4.33)] to achieve minimum meal frequency compared to those from the poorest households. Moreover, the contextual region emerged as a significant factor, with children residing in the city-based region being 1.75 times more likely [AOR = 1.75; 95%CI: (1.10, 2.77)] to meet minimum meal frequency standards compared to their counterparts in agrarian regions of Ethiopia.

### Random effects (measures of variation)

The study aimed to evaluate if the characteristics of clusters where 6–23-month-old children lived affected their minimum meal frequency. Model four, selected for its lowest Deviance Information Criterion (DIC), lowest Akaike Information Criterion (AIC), and highest log-likelihood ratio, provided the best explanation for minimum meal frequency. The null model revealed an Intra-class Correlation Coefficient (ICC) of 15.4%, indicating that around 15.4% of the variation in minimum meal frequency was linked to community-level factors, with significant variation across communities (clusters) at a *p*-value of <0.001.

After adjusting for individual and community-level factors, the final model maintained statistically significant variation in minimum meal frequency across communities (ICC = 8.1% and variance = 0.29). The final model showed a higher Proportional Change in Variance (PCV) of 50.8%, indicating that 50.8% of the variation in minimum meal frequency was explained by combined factors at both individual and community levels. Both the null model and the final model exhibited significant Median Odds Ratios (MOR). In the null model, the MOR was 2.07, while in the final model, it reduced to 1.67. This implies that 34% of the variation was explained by individual and community-level factors, leaving some unexplained variability in the final model (MOR = 1.67) (Table 6).

**Table 6:** Measure of variations and model fitness.

Characters		Model 1	Model 2	Model 3	Model 4
Random effect	Variance	0.59	0.42	0.28	0.29
Measure of variation	ICC	15.4%	11.4%	7.9%	8.1%
	PCV	reference	28.8%	50.5%	50.8%*
	MOR	2.07	1.85	1.65	1.67*
Model diagnostics	Log-likelihood	−807.28	−760.47	−772.05	−749.66*
	DIC	1614.56	1520.94	1544.1	1499.32*
	AIC	1618.57	1576.94	1568.11	1565.33*

*Model four is chosen due to superior fitted statistics of the log-likelihood ratio. Here’s a breakdown of the key model selections: Model 1 represents the null model without independent variables, Model 2 includes only individual-level variables, Model 3 comprises solely community-level variables, and Model 4 incorporates both individual and community-level variables. The evaluation metrics utilized include PCV (Proportional Change in Variance), ICC (Intra Class Correlation), MOR (Median Odds Ratio), AIC (Akaike Information Criteria), and DIC (Deviance Information Criteria).

## Discussion

According to the 2019 EMDHS data, 55.9% (95% CI: 53.2, 58.6) of breastfeeding children aged 6–23 months were fed with minimum meal frequency standards appropriate for their age and breastfeeding status. The finding has shown some improvement from the previous (2016) demographic health survey report (26). Furthermore, the finding was lower than the 2022 Kenyan and Nepal demographic health survey key indicator report (27, 28), however, it was higher than the 2022 Tanzanian, Ghana, Rwanda, and Liberia demographic health survey findings (29–32) and in line with the 2021 Gambian demographic health survey (33). The discrepancies in minimum meal frequency among different African countries might be due to various factors such as socioeconomic disparities which lead to diverse economic statuses, ranging from low-income to middle-income nations. Economic disparities can impact food access and affordability, and thus, meal frequency influences dietary patterns and eating habits between countries (34).

Cultural norms regarding meal timing, portion sizes, and food choices may also contribute to variations in minimum meal frequency. Disparities in nutrition education and health policies can affect meal frequency recommendations and dietary practices. Access to nutritional information, awareness of dietary guidelines, and implementation of public health interventions vary among countries, influencing eating habits (35).

The mixed effect analysis of EMDHS data revealed that delayed breastfeeding initiation time had a negative association with minimum meal frequency for 6–23 month breastfeeding children, this finding was consistent with the studies done in Amibara District (36). Another study also suggested that infants who initiated breastfeeding within the first hour of life exhibited more frequent feeding behaviors and had shorter intervals between feeds compared to those who initiated breastfeeding later (37). The World Health Organization (WHO) recommends early initiation of breastfeeding as an essential part of newborn care (38). Yet, this practice is far from universal. According to a study published in *The Lancet* (39), only about half of infants worldwide are breastfed within the first hour of birth.

This finding suggests that timely initiation of breastfeeding contributes to more regular and frequent feeding patterns in children in their later life. Timely initiation of breastfeeding and the establishment of regular feeding patterns have important implications for infant health and development (37). Studies have shown that infants who receive early and frequent breastfeeding have lower rates of infections, better growth outcomes, and improved cognitive development which is important to feed the child more frequently (40). These long-term benefits underscore the importance of promoting the timely initiation of breastfeeding and supporting breastfeeding practices that ensure adequate meal frequency for infants (37, 40).

Maternal education to at least primary level and higher level exhibited a positive association with minimum meal frequency compared with women without formal education. The finding was concurrent with studies conducted in Gambia (21), in Malawi (41), in Ghana (42) and different parts of Ethiopia (8, 18, 19, 43). This is because maternal education is often associated with increased knowledge about child nutrition and feeding practices (42, 44) and mothers or parents with education tend to be more receptive to acquiring new knowledge, have a greater understanding of the importance of proper child-feeding practices, and are able to adjust their behaviors more swiftly. In contrast, those without literacy skills may be less adaptable, more resistant to change (21).

Mothers with higher education levels are more likely to be aware of the importance of regular meals and balanced nutrition for their children (42) and more likely to have the knowledge and financial means to provide regular and adequate meals for their children (19, 44, 45), in contrast less educated mothers may not understand the need to feed children smaller meals more frequently, or they may not be aware of how to prepare age-appropriate, nutritious meals. Moreover, maternal education often serves as a proxy for socioeconomic status, with higher education levels associated with greater access to resources, including nutritious food options (45). These findings implies the importance of maternal education in promoting optimal feeding practices and improving child nutrition outcomes.

Household wealth index demonstrated a positive association, as children from the wealthiest households were more likely to achieve minimum meal frequency compared to those from the poorest households. This finding was consistent with studies conducted elsewhere in Ethiopia (7, 36, 46, 47), the study in Benin (16), the study in Uganda (48), and the study in Gambia (21). This was due to families with higher wealth levels typically having greater financial resources to purchase food, including diverse and nutritious options. As a result, children from the wealthiest households tend to have better access to regular meals with higher frequency (21).

Furthermore, the household wealth index serves as a proxy for socioeconomic status, which has a profound impact on dietary quality and meal frequency (45). Families with higher wealth levels are more likely to afford nutrient-rich foods and maintain regular meal patterns. This finding implies that the importance of addressing socioeconomic disparities can promote optimal child feeding practice and meal frequency.

Contextual regions emerged as a significant factor, with children residing in city-based regions were more likely to meet minimum meal frequency standards compared to agrarian communities. The finding of this study was supported by other studies conducted in Gambia (21), Ghana (42), Malawi (41) and Benin (16). City-based communities are more likely to feed their children with minimum meal frequency compared to agrarian communities due to factors such as greater access to food resources, higher income levels, cultural influences, availability of infrastructure, and public services, access to information on child feeding practice (42). These differences highlight the importance of addressing contextual factors in promoting optimal child nutrition and health outcomes across different context.

### Strength and limitation of the study

This study has several strengths, including the use of nationally representative data and a multilevel approach to identify both individual and community-level factors influencing Minimum meal frequency (MMF). This comprehensive approach offers valuable insights for developing targeted intervention strategies. The analysis also accounted for the national population through proper adjustments, such as weighting and consideration of the sample design.

However, the study is not without limitations. As the data were drawn from a cross-sectional survey, establishing the temporal relationships between variables was challenging, and there may have been recall bias due to the use of a 24-h recall method for measuring MMF. Furthermore, the study was limited to variables collected by the EMDHS, excluding important factors like the child’s health status, the mother’s employment, and her knowledge and perceptions about meal frequency. Lastly, since community-level factors influencing MMF had not been previously explored, it is difficult to compare these findings with other studies.

### Areas for further research

Further research is needed to explore variables such as the child’s health status, the mother’s employment status, her knowledge and perceptions about meal frequency, and other community-level factors.

## Conclusion and recommendation

The prevalence of minimum meal frequency (MMF) among 6–23-month breastfed children in Ethiopia was low. Both individual and community-level factors were significantly associated with MMF. Timing of breastfeeding initiation, maternal educational level, household wealth index and contextual regions were linked to MMF. The barriers to achieving MMF in Ethiopia are multifaceted, encompassing socio-economic factors. Overcoming these barriers, it is commendable to have comprehensive interventions, such as improving maternal education and increase awareness to IYCF key messages for mothers, promote early initiation of breast feeding, and addressing socio-economic barriers in households. Programs that enhance food security, empower women, and promote nutrition education are recommended to achieving MMF and improving child health outcomes in Ethiopia.

## Data Availability

The datasets presented in this study can be found in online repositories. The names of the repository/repositories and accession number (s) can be found: https://dhsprogram.com.

## Footnotes

^1^https://dhsprogram.com

## References

[1] OMS. WHO guideline for complementary feeding of infants and young children 6–23 months of age, vol. 2023. Geneva: WHO (2023).

[2] Ethiopian Public Health Institute (EPHI), ICF. Ethiopia Mini demographic and health Survey 2019: Final report [internet]. (2021). 1–207. Available at: https://dhsprogram.com/pubs/pdf/FR363/FR363.pdf (Accessed November 05, 2024).

[3] Acharya D Subedi R Lee K Yoo SJ Gautam S Singh JK. Correlates of the timely initiation of complementary feeding among children aged 6–23 months in rupandehi district, Nepal. Children. (2018) 5:1–9. doi: 10.3390/children5080106 PMC6111930 30082617

[4] Birhanu H Gonete KA Hunegnaw MT Aragaw FM. Minimum acceptable diet and associated factors among children aged 6-23 months during fasting days of orthodox Christian mothers in Gondar city, north West Ethiopia. BMC Nutr. (2022) 8:1–11. doi: 10.1186/s40795-022-00558-z, PMID: 35948943 PMC9364522

[5] Ahmed JA Sadeta KK Lembo KH. Complementary feeding practices and household food insecurity status of children aged 6–23 months in Shashemene City west Arsi zone, Oromia, Ethiopia. Nurs Res Pract. (2022) 2022:1–14. doi: 10.1155/2022/9387031, PMID: 35463294 PMC9019450

[6] UNICEF. Indicators for assessing infant and young child feeding practices. World Heal Organ [Internet]. (2010);WHA55 A55/:19. Available at: http://apps.who.int/iris/bitstream/handle/10665/44306/9789241599290_eng.pdf?sequence=1%0Ahttp://whqlibdoc.who.int/publications/2008/9789241596664_eng.pdf%5Cnhttp://www.unicef.org/programme/breastfeeding/innocenti.htm%5Cnhttp://innocenti15.net/declaration (Accessed October 28, 2024).

[7] Brhane E Grum T Abraha TH Aregawi G. Meal frequency and associated factors among children 6-23 months in Tahtay michew district. Res Sq. (2020):1–15. doi: 10.21203/rs.3.rs-70779/v1 PMC12880657 41650170

[8] Beyene M Worku AG Wassie MM. Dietary diversity, meal frequency and associated factors among infant and young children in Northwest Ethiopia: a cross-sectional study. BMC Public Health. (2015) 15:1–9. doi: 10.1186/s12889-015-2333-x 26433689 PMC4592571

[9] Union A. African regional nutritional strategy 2005–2015. African Union: Addis Ababa (2015).

[10] Hundstad S Ole P. Prevalence of child stunting in sub-Saharan Africa and its risk factors. Clin Nutr Open Sci. (2022) 42:49–61. doi: 10.1016/j.nutos.2022.01.009

[11] Anand RK. (ed.). Infant and young child feeding In: IAP textbook of pediatrics. Geneva: WHO Library Cataloguing-in-Publication Data. (2013). 127–7.

[12] Victora CG Adair L Fall C Hallal PC Martorell R Richter L. Maternal and child undernutrition: consequences for adult health and human capital. Lancet. (2008) 371:340–57. doi: 10.1016/S0140-6736(07)61692-4, PMID: 18206223 PMC2258311

[13] Feleke FW Mulaw GF. Minimum acceptable diet and its predictors among children aged 6-23 months in Mareka District, southern Ethiopia: community based cross-sectional study. Int J Child Heal Nutr. (2020) 9:202–11. doi: 10.6000/1929-4247.2020.09.04.7

[14] Kahssay M Ebrahim E Seid O Woldu E Reddy S. Infant and young child feeding practices and associated factors among children aged 0-23 months in Assayita District Afar region Ethiopia. J Food Nutr Sci. (2019) 7:96. doi: 10.11648/j.jfns.20190706.13

[15] Abebe H Gashu M Kebede A Abata H Yeshaneh A Workye H. Minimum acceptable diet and associated factors among children aged 6–23 months in Ethiopia. Ital J Pediatr. (2021) 47:1–10. doi: 10.1186/s13052-021-01169-3 34717712 PMC8557568

[16] Mitchodigni IM Amoussa Hounkpatin W Ntandou-Bouzitou G Avohou H Termote C Kennedy G. Complementary feeding practices: determinants of dietary diversity and meal frequency among children aged 6–23 months in southern Benin. Food Secur. (2017) 9:1117–30. doi: 10.1007/s12571-017-0722-y

[17] Farah S Derese T Abera L. Minimum acceptable diet and associated factors among children aged 6–23 months in jig-Jiga, Somali region, eastern Ethiopia, 2022. BMC Nutr. (2024) 10:1–12. doi: 10.1186/s40795-023-00740-x 38212859 PMC10785458

[18] Mekonnen TC Workie SB Yimer TM Mersha WF. Meal frequency and dietary diversity feeding practices among children 6–23 months of age in Wolaita Sodo town, southern Ethiopia. J Health Popul Nutr. (2017) 36:18. doi: 10.1186/s41043-017-0097-x, PMID: 28526058 PMC5437677

[19] Markos M Samuel B Challa A. Minimum acceptable diet and associated factors among 6–23 months old children enrolled in outpatient therapeutic program in the Tulla district, Sidama region, Ethiopia: a community-based cross-sectional study. J Health Popul Nutr. (2024) 43:1–9. doi: 10.1186/s41043-024-00581-9 38978134 PMC11232196

[20] Pervin N Macer D Lasker SP. Levels and determinants of complementary feeding pattern exclusive of minimum meal frequency and dietary diversity among children of 6 to 23 months in Bangladesh. Bangladesh J Bioeth. (2020) 9:28–44. doi: 10.3329/bioethics.v9i3.48924

[21] Terefe B Jembere MM Mekonnen BA. Minimum meal frequency practice and associated factors among children aged 6-23 months old in the Gambia: a multilevel mixed effect analysis. Sci Rep. (2023) 13:1–12. doi: 10.1038/s41598-023-49748-0, PMID: 38114621 PMC10730716

[22] Teshome F Tadele A. Trends and determinants of minimum acceptable diet intake among infant and young children aged 6-23 months in Ethiopia: a multilevel analysis of Ethiopian demographic and health survey. BMC Nutr. (2022) 8:1–11. doi: 10.1186/s40795-022-00533-8, PMID: 35513888 PMC9069791

[23] Tsegaw SA Dawed YA Amsalu ET. Exploring the determinants of exclusive breastfeeding among infants under-six months in Ethiopia using multilevel analysis. PLoS One. (2021) 16:e0245034–17. doi: 10.1371/journal.pone.0245034, PMID: 33439886 PMC7806157

[24] Hox J Moerbeek M van de Schoot R. (2017) Multilevel analysis techniques and applications, (3rd. ed.). New York: Routledge.

[25] Merlo J Chaix B Yang M Lynch J Råstam L. A brief conceptual tutorial of multilevel analysis in social epidemiology: linking the statistical concept of clustering to the idea of contextual phenomenon. J Epidemiol Community Health. (2005) 59:443–9. doi: 10.1136/jech.2004.023473 15911637 PMC1757045

[26] Chung SS Yeh CH Feng SJ Lai CS Yang JJ Chen CC. Demographic and health Survey Ethiopia. Proceedings of the International Symposium on the Physical and Failure Analysis of Integrated Circuits, IPFA. (2016). 279–282.

[27] KNBS. Kenya demographic and health Survey 2022. Demogr Heal Surv. (2023) 2022:1–23.

[28] Survey H. Ministry of Health, Nepal. New ERA, ICF Int Inc Nepal Demogr heal Surv 2022 Kathmandu, Nepal Minist Heal Popul 2022; Available at: https://dhsprogram.com/pubs/pdf/FR379/FR379.pdf (Accessed November 05, 2024).

[29] TDHS. Demographic and health Survey and malaria Indicator Survey. Pap Knowl Towar Media Hist Doc. (2022):1–23.

[30] GSS GHS and ICF. Ghana demographic and Health Survey 2022: key indicators report. Accra, Ghana, Rockville, Maryland, USA, GSS ICF. (2023); 5–24.

[31] National Institute of Statistics of Rwanda (NISR). Rwanda demographic and health Survey 2019–20 final report. Kigali, Rwanda, and Rockville, Maryland, USA: NISR and ICF (2021).

[32] Liberia Institute of Statistics and Geo-Information Services (LISGIS), Ministry of Health [Liberia] and I. Liberia demographic and health Survey 2019–20. Monrovia, Liberia and Rockville, Maryland, USA: Liberia Institute of Statistics and Geo-Information Services (LISGIS) (2021).

[33] Goldsmith MUD. The Gambia demographic and health Survey 2019-2020 Hip Hop around the World [2 volumes]: An Encyclopedia [2 volumes]. Gambia Bureau of Statistics Banjul, The Gambia DHS (2021).

[34] Goryakin Y Lobstein T James WPT Suhrcke M. Social science and medicine the impact of economic, political and social globalization on overweight and obesity in the 56 low and middle income countries. Soc Sci Med. (2015) 133:67–76. doi: 10.1016/j.socscimed.2015.03.030, PMID: 25841097 PMC4416723

[35] South Africa. National food and nutrition security plan for South Africa 2018–2023. (2017): 1–229.

[36] Wagris M Seid A Kahssay M Ahmed O. Minimum meal frequency practice and its associated factors among children aged 6–23 months in Amibara District, north East Ethiopia. J Environ Public Health. (2019) 2019:1–7. doi: 10.1155/2019/8240864, PMID: 31929807 PMC6942825

[37] Group NS. Timing of initiation, patterns of breastfeeding, and infant survival: prospective analysis of pooled data from three. Lancet Glob Heal. (2016) 4:e266–75. doi: 10.1016/S2214-109X(16)00040-1, PMID: 27013313

[38] Organization WH. Postnatal care of the mother and newborn 2013. World Heal Organ (2013); 1–72. Available at: http://apps.who.int/iris/bitstream/10665/97603/1/9789241506649_eng.pdf (Accessed October 28, 2024).

[39] Victora CG Bahl R Barros AJD França GVA Horton S Krasevec J. Breastfeeding in the 21st century: epidemiology, mechanisms, and lifelong effect. Lancet. (2016) 387:475–90. doi: 10.1016/S0140-6736(15)01024-7, PMID: 26869575

[40] Horta BL Loret De Mola C Victora CG. Long-term consequences of breastfeeding on cholesterol, obesity, systolic blood pressure and type 2 diabetes: a systematic review and meta-analysis. Acta Paediatr Internat J Paediatr. (2015) 104:30–7. doi: 10.1111/apa.13133 26192560

[41] Nkoka O Mhone TG Ntenda PAM. Factors associated with complementary feeding practices among children aged 6-23 mo in Malawi: an analysis of the demographic and health Survey 2015–2016. Int Health. (2018) 10:466–79. doi: 10.1093/inthealth/ihy047, PMID: 30052967

[42] Dadzie LK Amo-Adjei J Esia-Donkoh K. Women empowerment and minimum daily meal frequency among infants and young children in Ghana: analysis of Ghana demographic and health survey. BMC Public Health. (2021) 21:1–9. doi: 10.1186/s12889-021-11753-1 34535097 PMC8449451

[43] Gezahegn H Tegegne M. Magnitude and its predictors of minimum dietary diversity feeding practice among mothers having children aged 6–23 months in Goba town, Southeast Ethiopia, 2018: a community-based cross-sectional study. Nutr Diet Suppl. (2020) 12:215–22. doi: 10.2147/NDS.S243521

[44] Fadare O Amare M Mavrotas G Akerele D Ogunniyi A. Mother’s nutrition-related knowledge and child nutrition outcomes: empirical evidence from Nigeria. PLoS One. (2019) 14:1–17. doi: 10.1371/journal.pone.0215110 PMC6394922 30817794

[45] Choudhury S Headey DD. Economics and human biology household dairy production and child growth: evidence from Bangladesh. Econ Hum Biol. (2018) 30:150–61. doi: 10.1016/j.ehb.2018.07.001, PMID: 30048913 PMC6130515

[46] Belew AK Ali BM Abebe Z Dachew BA. Dietary diversity and meal frequency among infant and young children: a community based study. Ital J Pediatr. (2017) 43:6–15. doi: 10.1186/s13052-017-0384-6 28810887 PMC5558775

[47] Wake AD. Prevalence of minimum meal frequency practice and its associated factors among children aged 6–23 months in Ethiopia: a systematic review and Meta-analysis Global Pediatric Health. North America: Sage (2021). 8 p. 10.1177/2333794X211026184 PMC8226363 34235233

[48] Scarpa G Berrang-Ford L Galazoula M Kakwangire P Namanya DB Tushemerirwe F. Identifying predictors for minimum dietary diversity and minimum meal frequency in children aged 6–23 months in Uganda. Nutrients. (2022) 14:1–18. doi: 10.3390/nu14245208 PMC9786234 36558366

